# Supplementary material for: Comprehensive assessment of mRNA isoform detection methods for long-read sequencing data
Source: Nat Commun. 2024 May 10;15:3972. doi: 10.1038/s41467-024-48117-3 (PMC11087464; doi:10.1038/s41467-024-48117-3)
Supplement: Supplementary file 1 — Supplementary Information [file 41467_2024_48117_MOESM1_ESM.pdf]

## Supplementary Information

# Comprehensive Assessment of mRNA Isoform Detection Methods for Long-Read Sequencing Data

Yaqi Su[1,2,9], Zhejian Yu[1,2], Siqian Jin[1,2], Zhipeng Ai[3], Ruihong Yuan[2], Xinyi Chen[1,2], Ziwei Xue[1,2], Yixin Guo[1,2], Di Chen[4,5], Hongqing Liang[3], Zuozhu Liu[6], Wanlu Liu[1,2,7,8*]

1. Department of Orthopedic Surgery of the Second Affiliated Hospital, Zhejiang University School of Medicine, Zhejiang University, Hangzhou, Zhejiang 310009, China
2. Centre of Biomedical Systems and Informatics of Zhejiang University-University of Edinburgh Institute (ZJU-UoE Institute), International Campus, Zhejiang University, Haining, Zhejiang 314400, China
3. Division of Human Reproduction and Developmental Genetics, Women's Hospital, Zhejiang University School of Medicine, Zhejiang University, Hangzhou, Zhejiang 310006, China
4. Center for Reproductive Medicine of The Second Affiliated Hospital Zhejiang University School of Medicine, Zhejiang University, Hangzhou, Zhejiang, 310009, China
5. Centre for Regeneration and Cell Therapy of Zhejiang University-University of Edinburgh Institute (ZJU-UoE Institute), International Campus, Zhejiang University, Haining, Zhejiang 314400, China
6. Zhejiang University-Angel Align Inc. R&D Center for Intelligent Healthcare, Zhejiang University-University of Illinois at Urbana-Champaign Institute (ZJU-UIUC Institute), International Campus, Zhejiang University, Haining, Zhejiang 314400, China
7. Future Health Laboratory, Innovation Center of Yangtze River Delta, Zhejiang University, Jiaxing, 314100, China
8. Alibaba-Zhejiang University Joint Research Center of Future Digital Healthcare, Zhejiang University, Hangzhou, Zhejiang 310058, China
9. Current Address: Department of Molecular and Cell Biology, University of California, Berkeley, CA, 94720, USA

* Corresponding author. Email: wanluliu@intl.zju.edu.cn

# Supplementary Figure 1

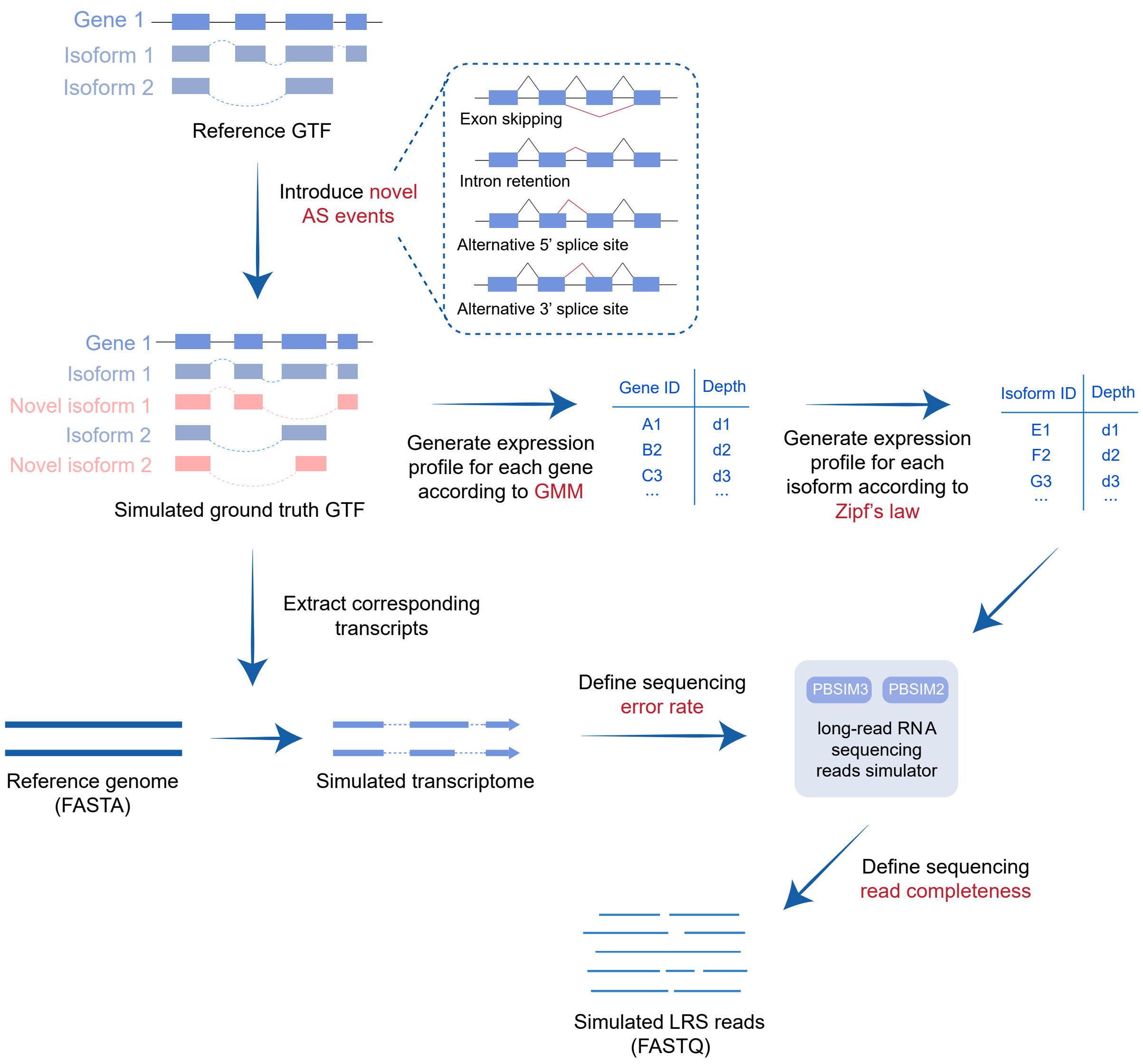


**Supplementary Figure 1. Schematic Workflow for YASIM.**
The workflow of YASIM involves several steps to generate simulated long-read RNA-seq data. (1) Introduction of novel AS events: Four types of novel AS events are introduced into the original reference annotation GTF, creating a simulated ground truth GTF with different isoform information. (2) Generation of read depth: Read depth is generated for each simulated gene using a Gaussian mixture model (GMM). The read depth for each isoform within a gene is simulated based on Zipf's law. (3) Extraction of simulated transcriptome: The simulated transcriptome is extracted from the provided reference genome FASTA file, based on the simulated ground truth GTF. (4) Generation of long-read RNA-seq reads: The simulated transcriptome and the generated expression profile are fed into the LRS reads generator, PBSIM2 and PBSIM3. These tools transform the entire transcriptome into long-read RNA-seq reads. (5) Simulation of sequencing errors: The long-read RNA-seq reads are subjected to simulation of sequencing errors according to a user-defined error model and error rate. (6) Adjustment of read completeness: The read completeness is adjusted based on user-defined parameters.

# Supplementary Figure 2

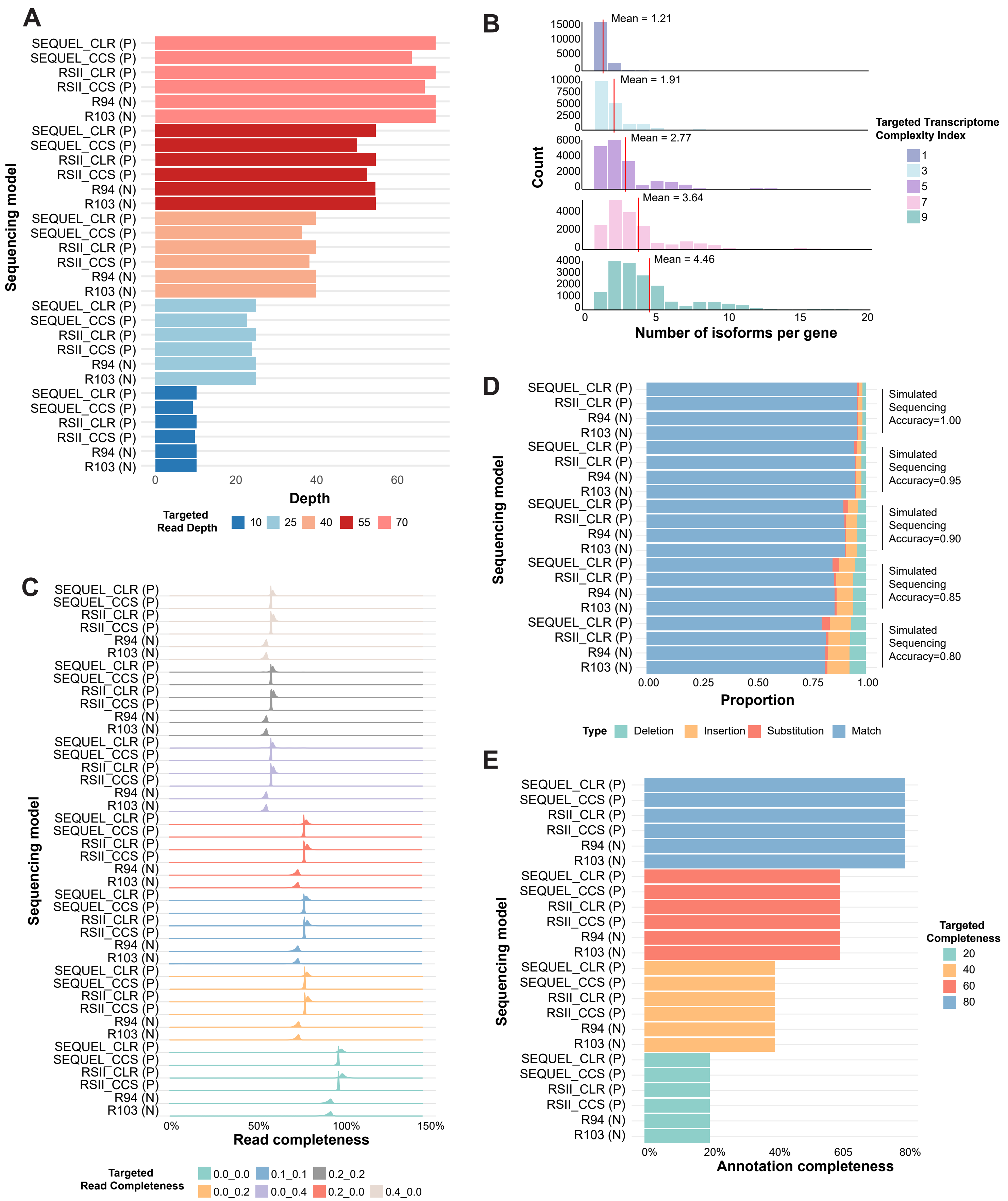


**Supplementary Figure 2. Quality Control for Simulated Data.**
**A.** Mean read depth of each simulated dataset generated with a targeted expression profile. **B.** Number of isoforms per gene for simulated data with different transcriptome complexity indexes. **C.** Read completeness of guidance annotation extracted according to the targeted annotation completeness (0.0_0.0: 100% complete, 0.1_0.1: 10% truncated from both ends; 0.2_0.2: 20% truncated from both ends; 0.2_0.0: 20% truncated from 5' end; 0.4_0.0: 40% truncated from 5' end; 0.0_0.2: 20% truncated from 3' end; 0.0_0.4: 40% truncated from 3' end). **D.** Error profiles for each simulated dataset obtained with the corresponding targeted accuracy. **E.** Distribution of read completeness for each dataset simulated with targeted read completeness. All panels were visualized using the second replicate of simulated data. N and P represent datasets generated from the Nanopore and Pacbio platforms, respectively. Source data underlying A, B, D, and E are provided as a Source Data file.

# Supplementary Figure 3

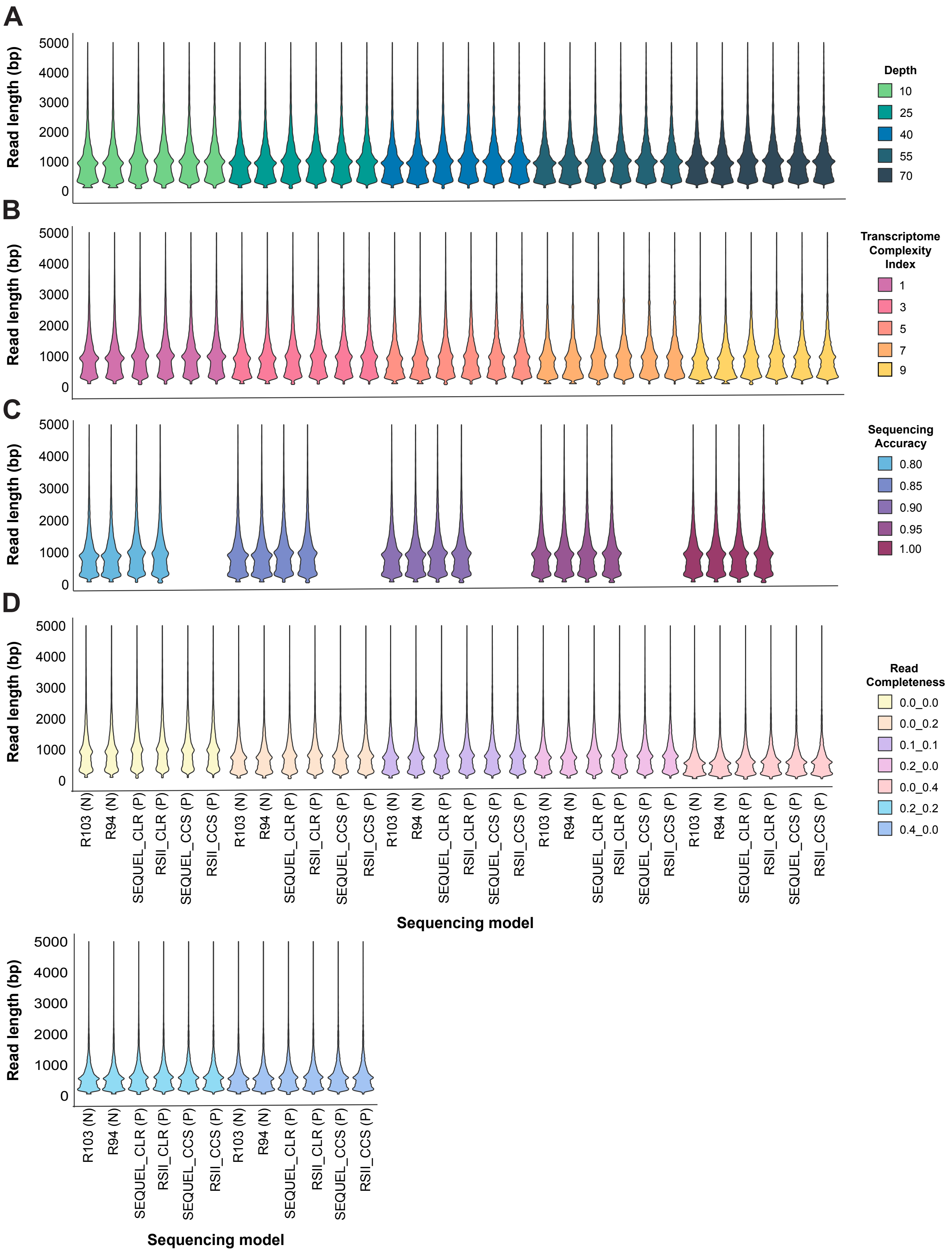


**Supplementary Figure 3. Read Length Distribution of Simulated Data.**
**A-D.** The distribution of read lengths for datasets simulated with different read depths (A), genome complexity index (B), sequencing accuracy (C), and read completeness (D). Reads over 5000 base pairs were filtered out in this analysis. N and P represent datasets generated from the Nanopore and Pacbio platforms, respectively.

# Supplementary Figure 4

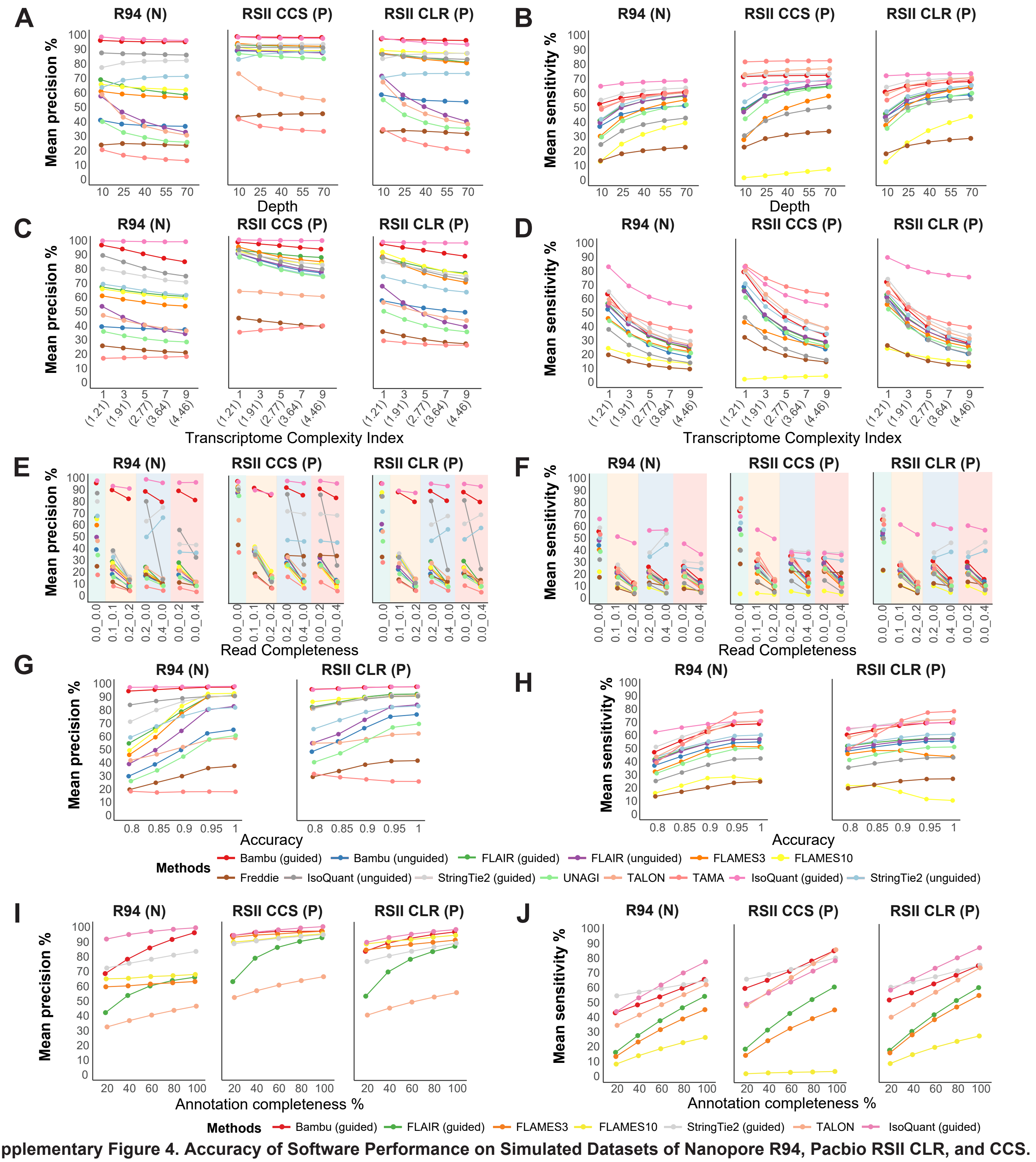


**Supplementary Figure 4. Accuracy of Software Performance on Simulated Datasets of Nanopore R94, Pacbio RSII CLR, and CCS. A, B.** Precision (A) and sensitivity (B) of the tested methods on simulated data from Nanopore R94, Pacbio RSII CLR, and CCS across varying read depths (10X, 25X, 40X, 55X, 70X, with three replicates for each sequencing platform, totaling n=45). **C, D.** Precision (C) and sensitivity (D) of the performance of tested methods obtained on simulated data of Nanopore R103, Pacbio SEQUEL CLR, and CCS across different transcriptome complexity indices (1, 3, 5, 7, 9; values in parentheses denote the actual mean number of isoforms per gene simulated), with three replicates per sequencing platform (n=45 in total). **E, F.** Precision (E) and sensitivity (F) of the performance of tested methods obtained on simulated data of Nanopore R94, Pacbio RSII CLR, and CCS with different read completeness (0.0_0.0: 100% complete, 0.1_0.1: 10% truncated from both ends; 0.2_0.2: 20% truncated from both ends; 0.2_0.0: 20% truncated from 5' end; 0.4_0.0: 40% truncated from 5' end; 0.0_0.2: 20% truncated from 3' end; 0.0_0.4: 40% truncated from 3' end, three replicates for each sequencing platform, n=63 in total). **G, H.** Precision (G) and sensitivity (H) of the performance of tested methods on simulated data of Nanopore R94, Pacbio RSII CLR, and CCS with different read accuracy (0.8, 0.85, 0.9, 0.95, 1, three replicates for each sequencing platform, n=30 in total). **I, J.** Precision (I) and sensitivity (J) of the performance of tested methods on simulated data of Nanopore R94, Pacbio RSII CLR, and CCS with different annotation completeness (20%, 40%, 60%, 80%, 100%, three replicates for each sequencing platform, n=45 in total). N and P represent datasets generated from the Nanopore and Pacbio platforms, respectively. All reported values are expressed as means, with Standard Deviation (SD) detailed in the Source Data file.  Source data underlying A-J is provided as a Source Data file.

# Supplementary Figure 5

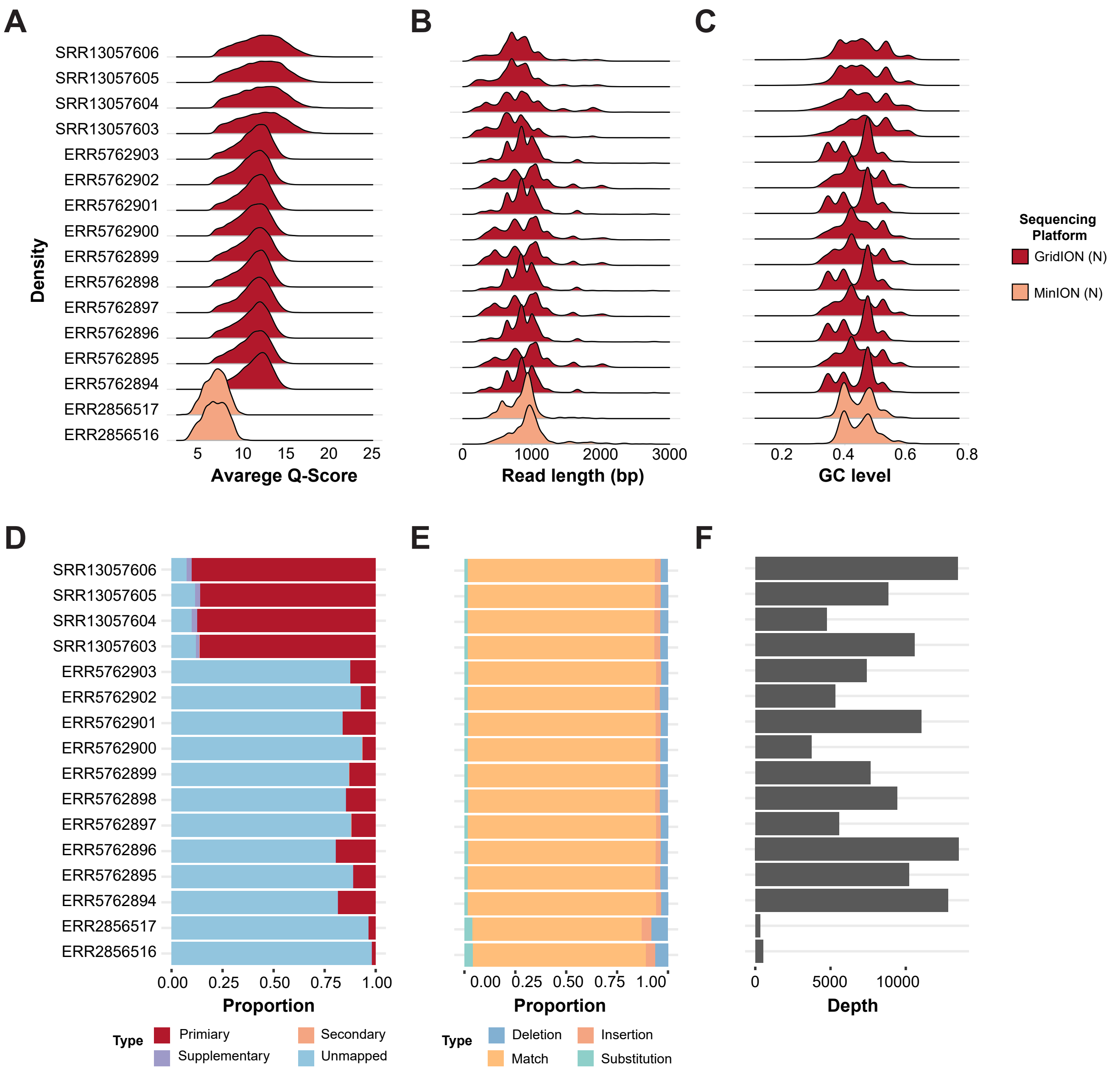


**Supplementary Figure 5. Quality Control for Sequins Datasets.**
**A-F.** Quality control analysis of Sequins long-read RNA-seq datasets was performed on average read quality score (Q-Score from FASTQ file) distribution (A), read length (B), GC level distribution (C), read alignment status (D), type of basel-evel matches and mismatches (E), and read depths (F). Source data underlying D and F are provided as a Source Data file.

# Supplementary Figure 6

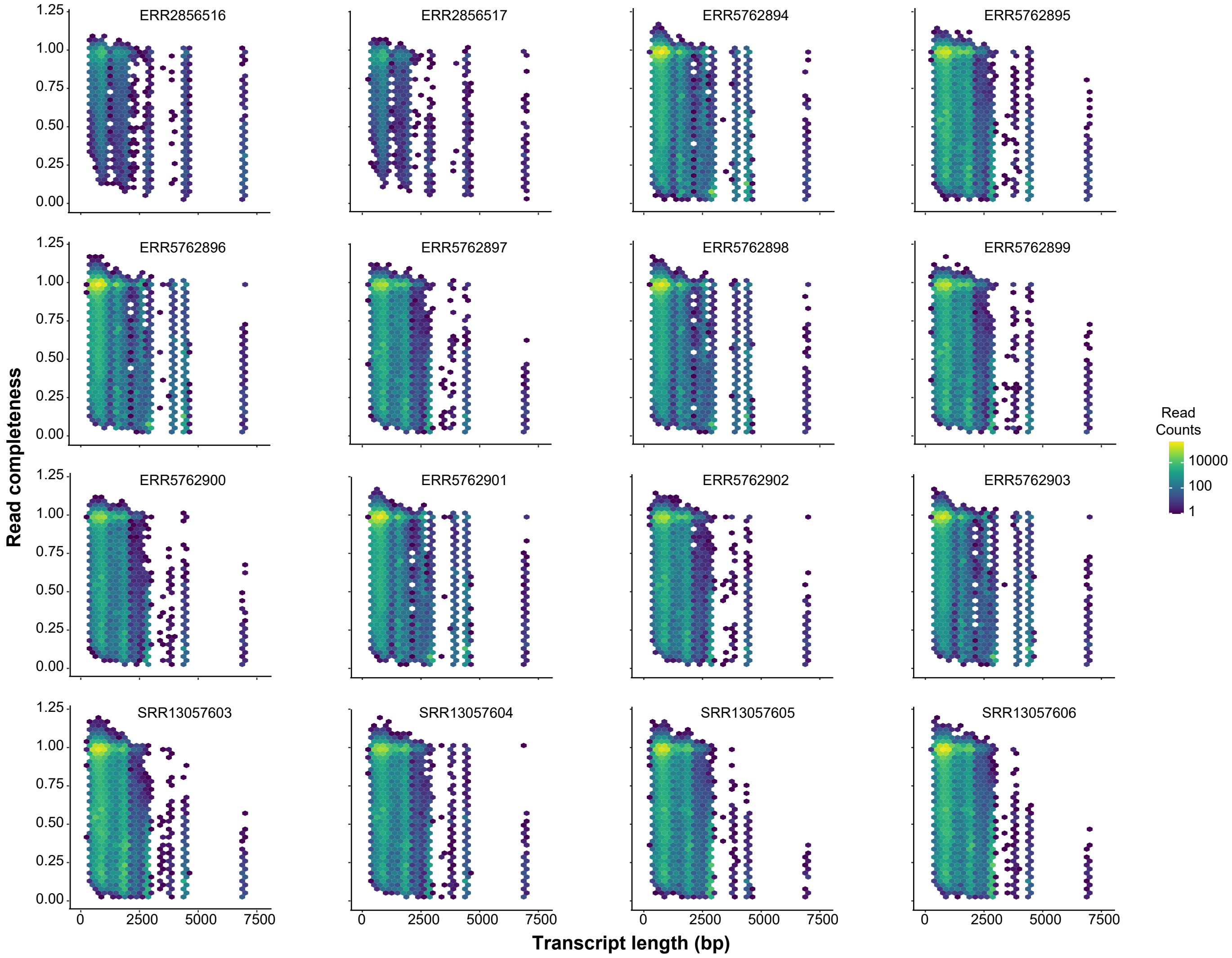


**Supplementary Figure 6. Read Completeness Distribution of Sequins Datasets.**
Distribution of read completeness and transcript length for different Sequins long-read RNA-seq datasets.

# Supplementary Figure 7

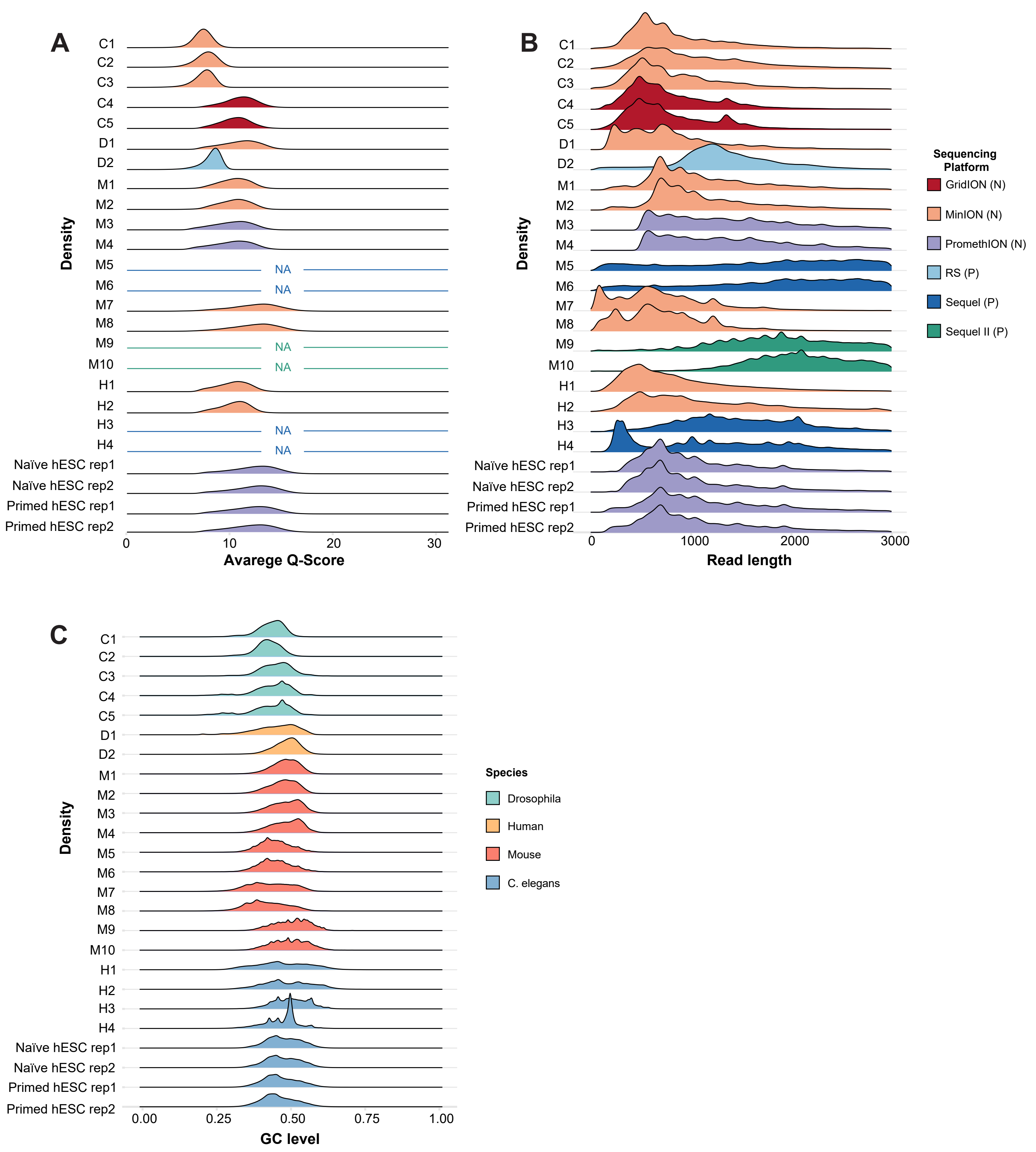


**Supplementary Figure 7. GC Level, Read length, and Read Quality Distribution of Real Datasets.**
**A-C.** Quality control analysis of real datasets was performed on average read quality score (Q-Score from FASTQ file) distribution (A), read length (B), and GC level distribution (C). N.A. in A represents Q-Score not available. The publicly available real datasets originate from the following sources: C1: L1 larval stage of C. elegans, C2: mix stage of C. elegans, C3: young adult stage of C. elegans; C4: Wild type C. elegans total RNA replicate 1, C5: Wild type C. elegans total RNA replicate 2, D1: Drosophila, D2: Drosophila testis, M1: mouse activated CD8 T cell, M2: mouse naïve CD8 T cell, M3: mouse retinal cells (control), M4: mouse retinal cells (glaucomatous), M5: mouse CD4SP cells, M6: mouse CD8SP cells, M7: mouse neural stem cells (E15.5), M8: mouse neural stem cells (P1.5), M9: mouse cerebral cells, M10: mouse hippocampus cells. H1: human Beta cells, H2: human Beta cells treated with cytokines, H3: human Hela cells, H4: human iPSC cells. The long-read RNA-seq dataset on Naïve and Primed hESCs was generated in this study.

# Supplementary Figure 8

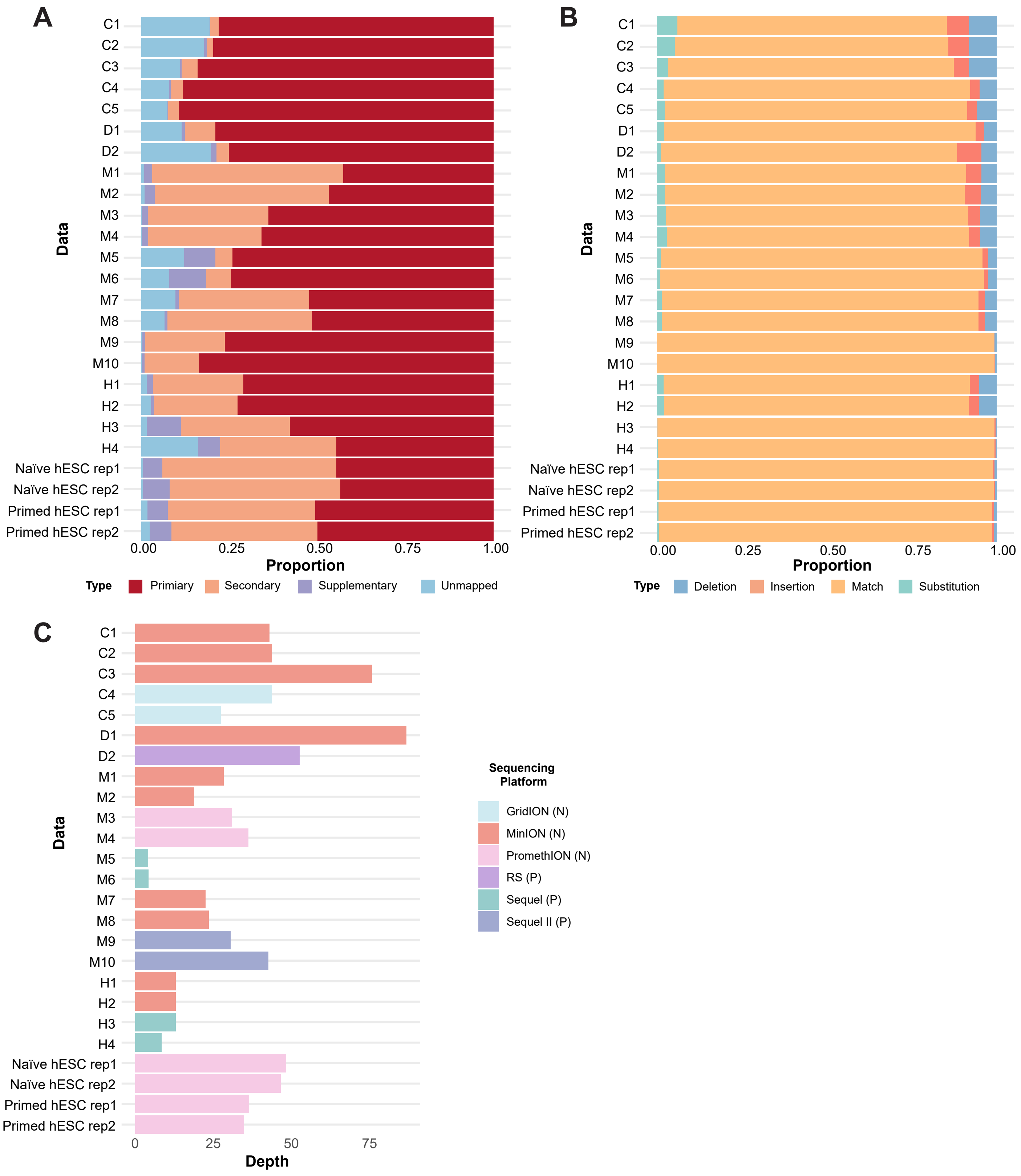


**Supplementary Figure 8. Mapping Status, Error Rate Distribution, and Read Depths of Real Datasets.**
**A-C.** Quality control analyses of real datasets was performed to assess the read alignment status (A), type of basel-evel matches and mismatches (B), and read depths (C).The publicly available real datasets originate from the following sources: C1: L1 larval stage of C. elegans, C2: mix stage of C. elegans, C3: young adult stage of C. elegans; C4: Wild type C. elegans total RNA replicate 1, C5: Wild type C. elegans total RNA replicate 2, D1: Drosophila, D2: Drosophila testis, M1: mouse activated CD8 T cell, M2: mouse naïve CD8 T cell, M3: mouse retinal cells (control), M4: mouse retinal cells (glaucomatous), M5: mouse CD4SP cells, M6: mouse CD8SP cells, M7: mouse neural stem cells (E15.5), M8: mouse neural stem cells (P1.5), M9: mouse cerebral cells, M10: mouse hippocampus cells. H1: human Beta cells, H2: human Beta cells treated with cytokines, H3: human Hela cells, H4: human iPSC cells. The long-read RNA-seq dataset on Naïve and Primed hESCs was generated in this study. Source data underlying A-C are provided as a Source Data file.

# Supplementary Figure 9

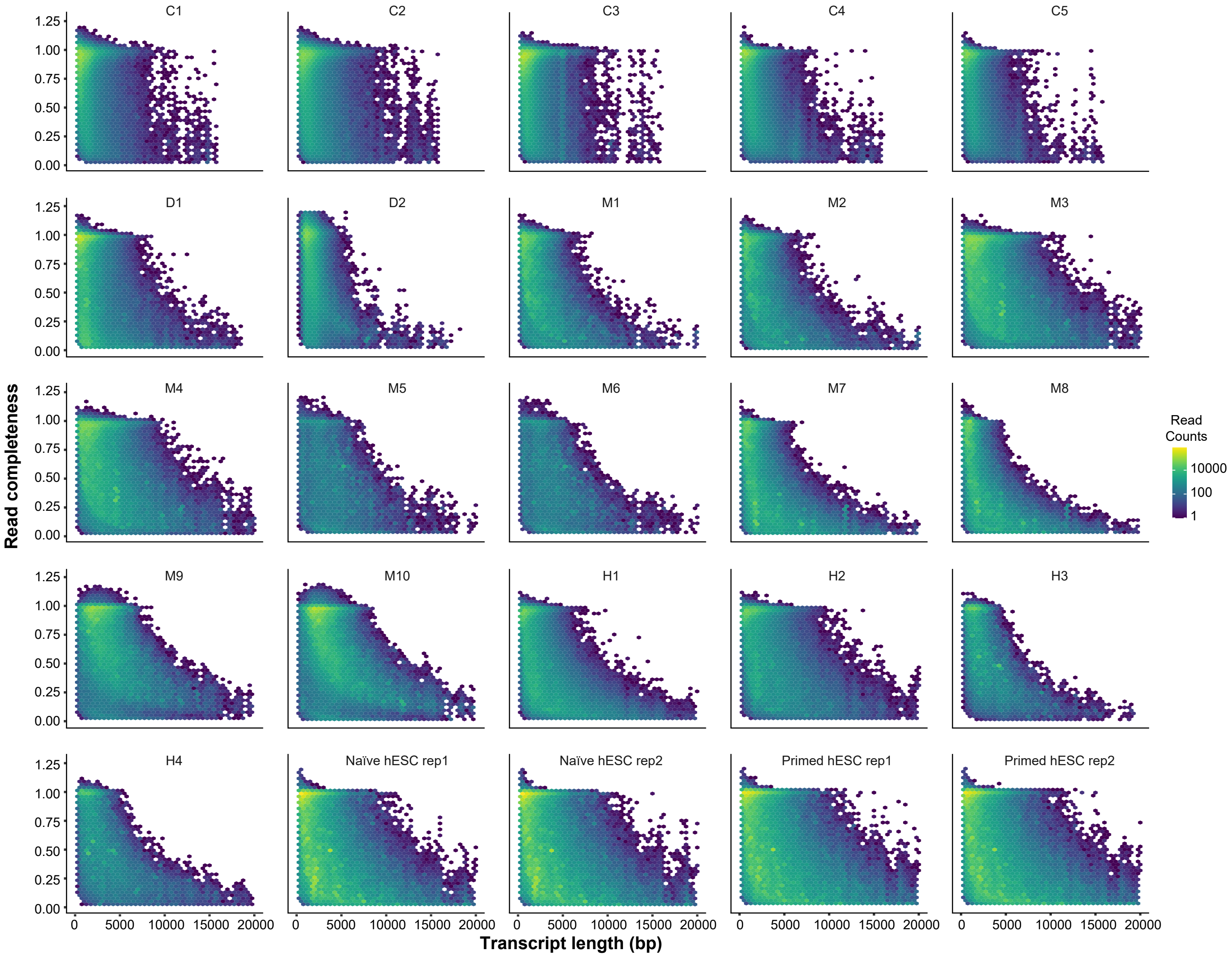


**Supplementary Figure 9. Read Completeness Distribution of Real Datasets.**
Distribution of read completeness and transcript length for different real datasets. The publicly available real datasets originate from the following sources: C1: L1 larval stage of C. elegans, C2: mix stage of C. elegans, C3: young adult stage of C. elegans; C4: Wildtype C. elegans total RNA replicate 1, C5: Wildtype C. elegans total RNA replicate 2, D1: Drosophila, D2: Drosophila testis, M1: mouse activated CD8 T cell, M2: mouse naïve CD8 T cell, M3: mouse retinal cells (control), M4: mouse retinal cells (glaucomatous), M5: mouse CD4SP cells, M6: mouse CD8SP cells, M7: mouse neural stem cells (E15.5), M8: mouse neural stem cells (P1.5), M9: mouse cerebral cells, M10: mouse hippocampus cells. H1: human Beta cells, H2: human Beta cells treated with cytokines, H3: human Hela cells, H4: human iPSC cells. The long-read RNA-seq dataset on Naïve and Primed hESCs was generated in this study.

# Supplementary Figure 10

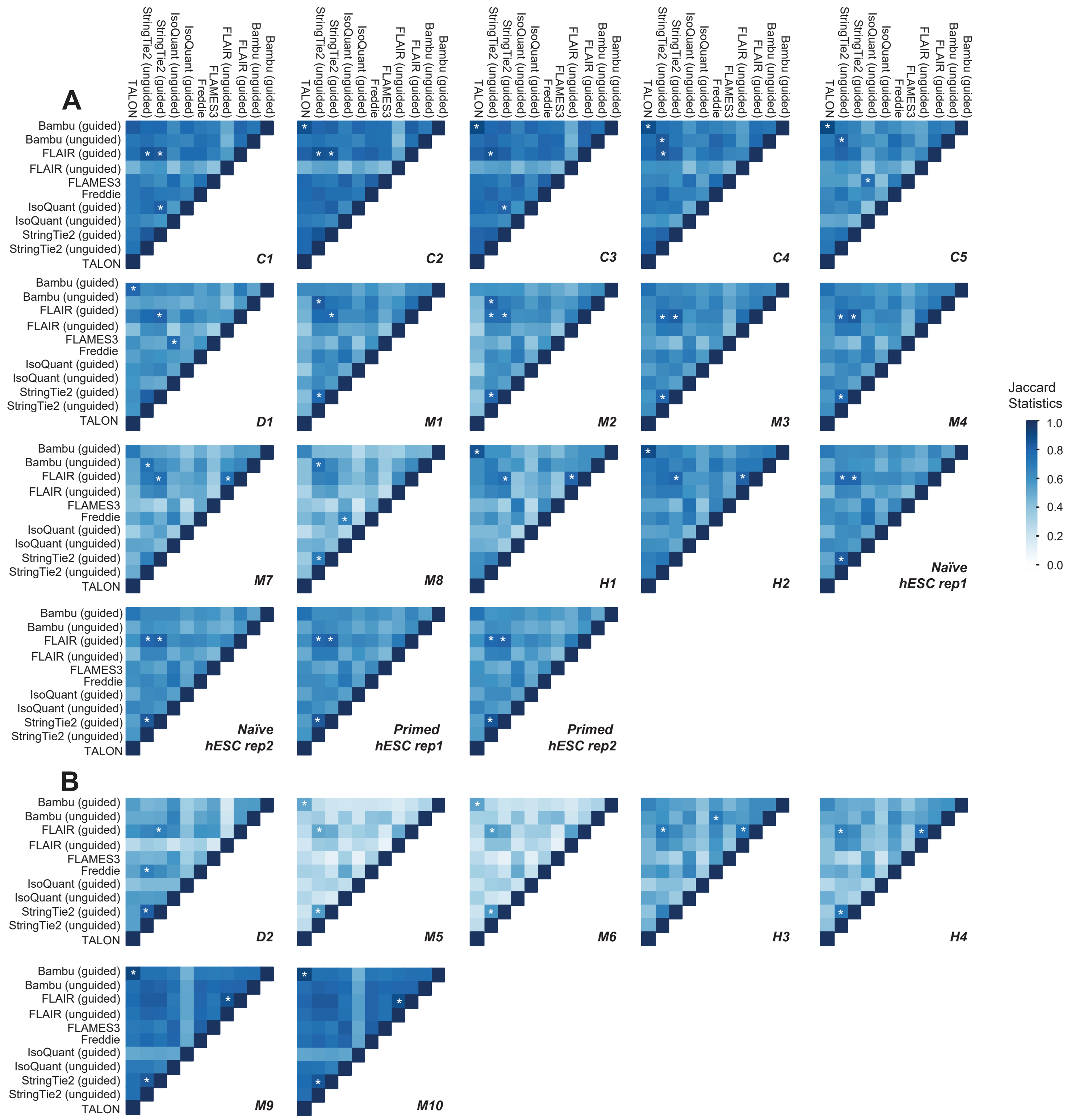


**Supplementary Figure 10. Similarity Analyses with Real Datasets.**

**A, B.** Heatmap showing pairwise Jaccard statistics representing the overlap of isoforms identified by different methods across 25 real datasets collected using the Nanopore (A) or Pacbio platform (B). The * symbol denotes the top three Jaccard overlaps in each dataset. The publicly available real datasets originate from the following sources: C1: L1 larval stage of C. elegans, C2: mix stage of C. elegans, C3: young adult stage of C. elegans; C4: Wild type C. elegans total RNA replicate 1, C5: Wild type C. elegans total RNA replicate 2, D1: Drosophila, D2: Drosophila testis, M1: mouse activated CD8 T cell, M2: mouse naïve CD8 T cell, M3: mouse retinal cells (control), M4: mouse retinal cells (glaucomatous), M5: mouse CD4SP cells, M6: mouse CD8SP cells, M7: mouse neural stem cells (E15.5), M8: mouse neural stem cells (P1.5), M9: mouse cerebral cells, M10: mouse hippocampus cells. H1: human Beta cells, H2: human Beta cells treated with cytokines, H3: human Hela cells, H4: human iPSC cells. The long-read RNA-seq dataset on Naïve and Primed hESCs was generated in this study.

# Supplementary Figure 11

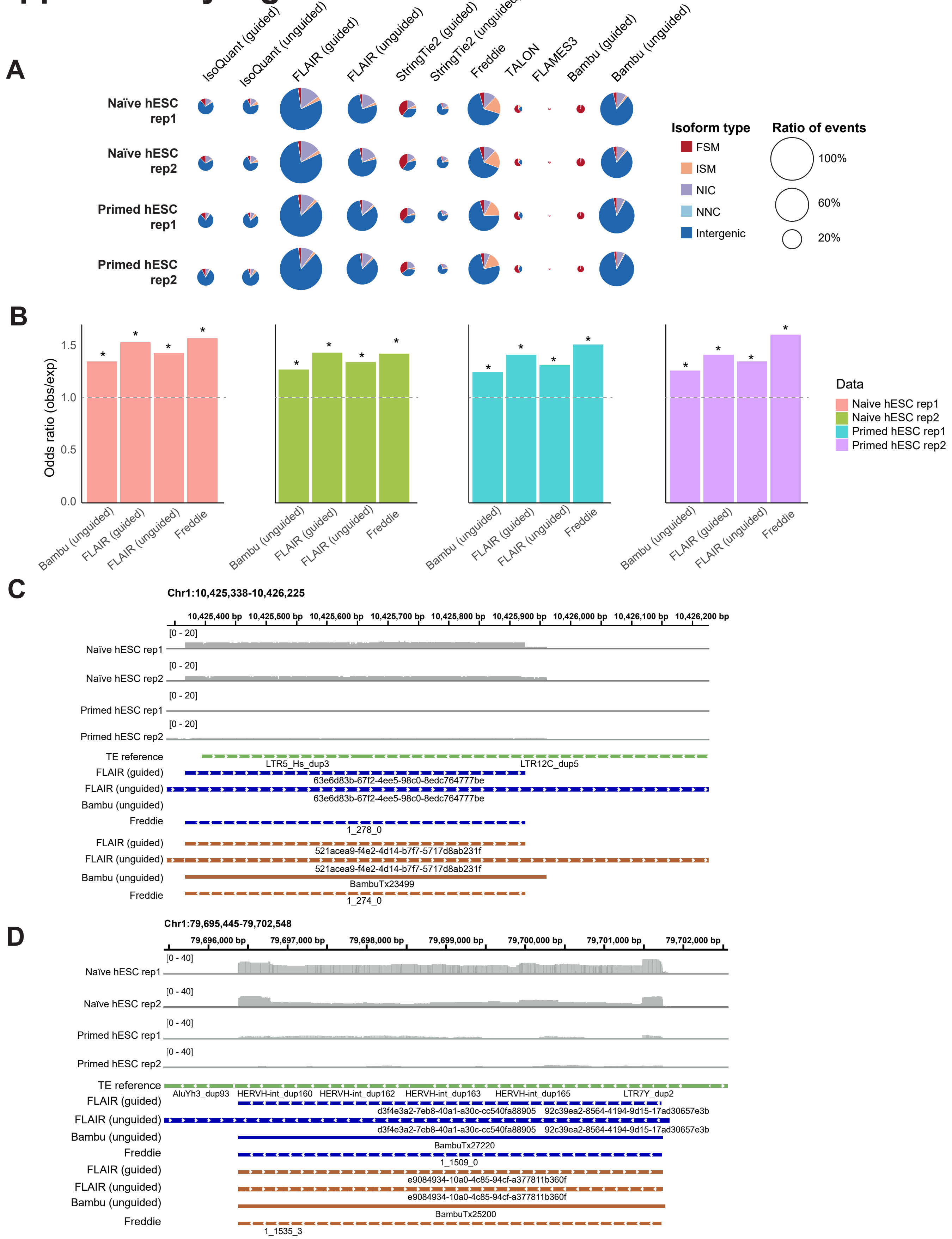


**Supplementary Figure 11. Isoform Classification for Mono-exonic Transcripts.**

**A.** Pie chart showing the different types of mono-exonic isoforms detected by different methods in Naïve and Primed hESC long-read RNA-seq datasets. FSM, ISM, NIC, NNC represent full splice match, incomplete splice match, novel in catalog, and novel not in catalog, respectively. **B.** The odds ratio (observed over expected) of overlapping regions between TEs and intergenic mono-exon transcripts identified by Bambu (unguided), FLAIR (guided), FLAIR (unguided), Freddie compared with randomly shuffled intergenic regions. **C, D.** IGV screenshots displaying Naïve and Primed hESC long-read RNA-seq tracks over representative transposable element sites, specifically LTR5_Hs_dup3 (B) and LTR7Y_dup2 (C). The green bar represents the reference annotation for transposable elements, blue bars represent the mono-exonic transcripts identified by FLAIR (guided), FLAIR (unguided), Bambu (unguided), and Freddie in Naïve hESC rep1, and brown bars represent the mono-exonic transcripts identified by FLAIR (guided), FLAIR (unguided), Bambu (unguided), and Freddie in Naïve hESC rep2. The text under each bar represents the transcript ID assigned by different methods, and the white arrows on each bar represent the annotated transcript direction by each method. Statistical analysis was performed with the Fisher exact test (**P* < 0.05). Source data underlying A and B are provided as a Source Data file.

# Supplementary Figure 12

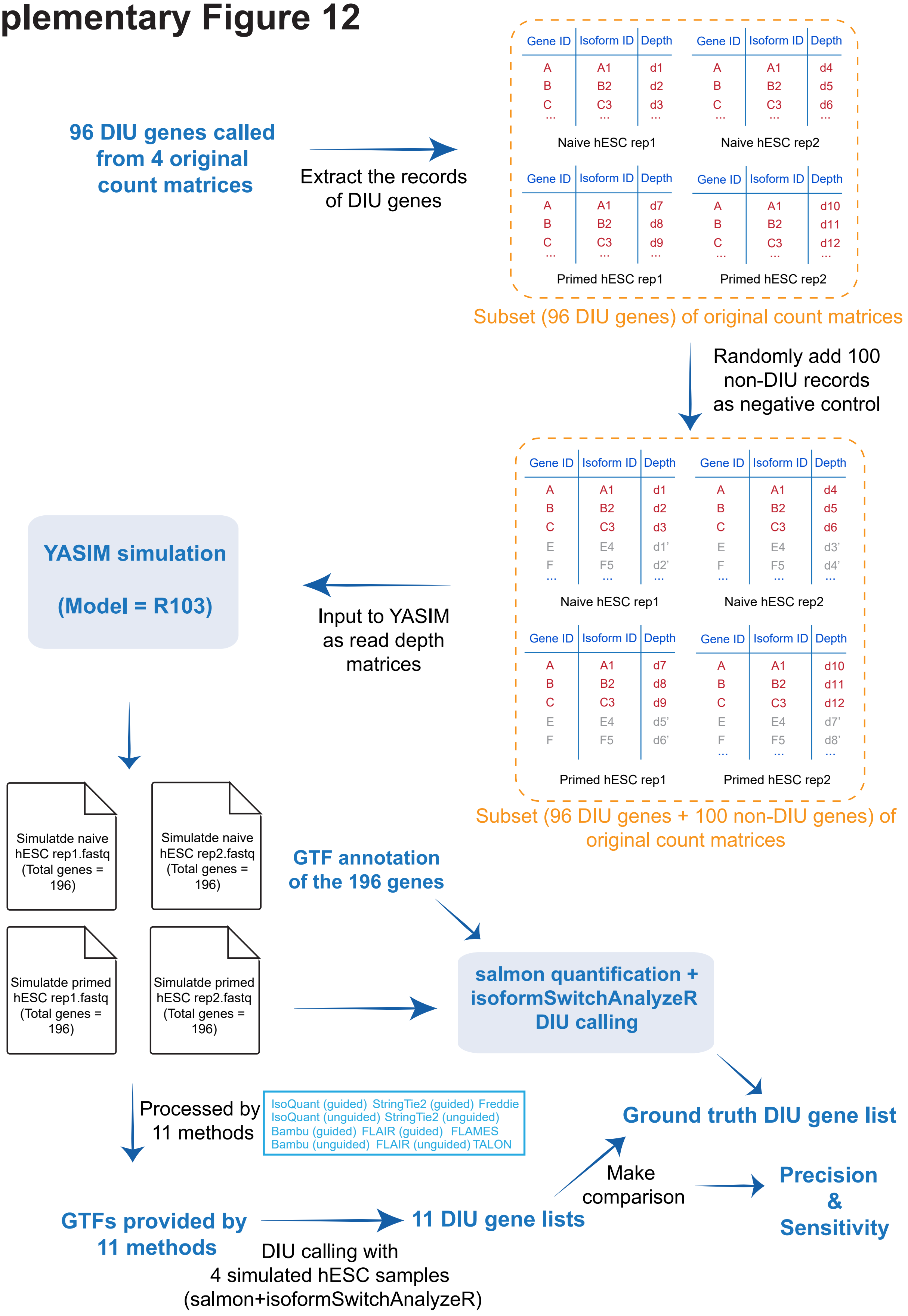


**Supplementary Figure 12. Schematic Workflow for DIU Simulation.**
This figure presents the process for simulating Differential Isoform Usage (DIU). Initially, 96 DIU genes were identified from the count matrices of actual Naïve and Primed hESC long-read RNA-seq datasets. Isoform counts for these genes were then extracted from the original count matrices. An additional 100 genes were randomly selected from the reference annotation, with the exclusion of the 96 DIU genes, and were incorporated into the count matrices. The resulting isoform count matrix, encompassing 196 genes, was used to create expression profiles in YASIM. Subsequently, four simulated long-read RNA-seq datasets were generat-ed employing the Nanopore R103 error model. These simulated reads were aligned and processed using eleven benchmarked methods, followed by salmon quantification and DIU calling using isoformSwitchAnalyzeR. Meanwhile, the corresponding GTF annotation of the 196 genes plus the four simulated datasets were also processed by salmon and isoformSwitchAnalyzeR, and the DIU gene list obtained were served as ground truth. The eleven resulting DIU gene lists were then compared with the ground truth DIUs to calculate the respective precision and sensitivity values.

# Supplementary Figure 13

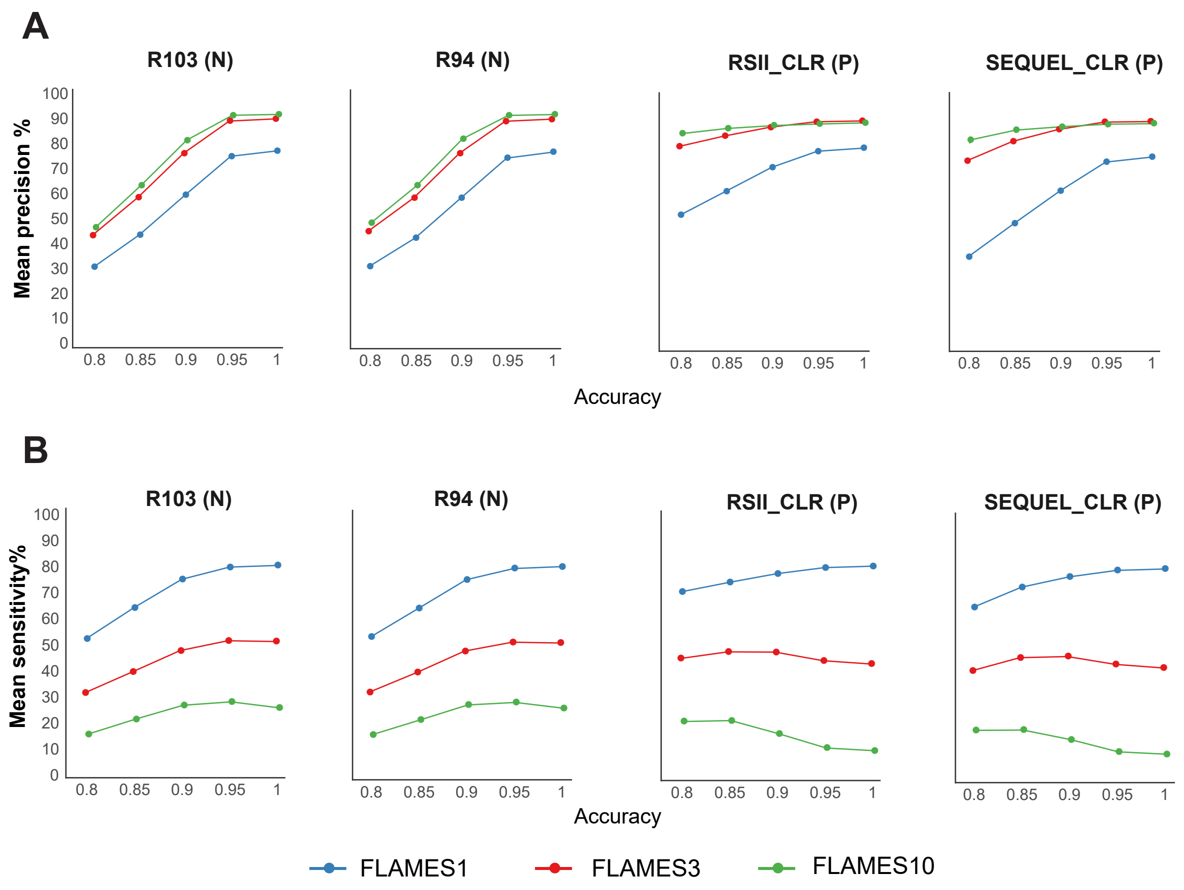


**Supplementary Figure 13. FLAMES with Different "min_supp_cnt" Setting under Different Read Accuracy Simulation Scenarios.**

**A, B.** Mean precision (A) and sensitivity (B) for FLAMES with "min_supp_cnt" set as 1 (FLAMES1), 3 (FLAMES), and 10 (FLAMES10) under different simulated datasets with read accuracy of 0.8, 0.85, 0.9, 0.95, 1 (n=60 in total). N and P represent datasets generated from the Nanopore and Pacbio platforms, respectively. All values are denoted as mean. Standard deviation (SD) is provided in the Source Data file. Source data underlying A and B are provided as a Source Data file.

# Supplementary Figure 14

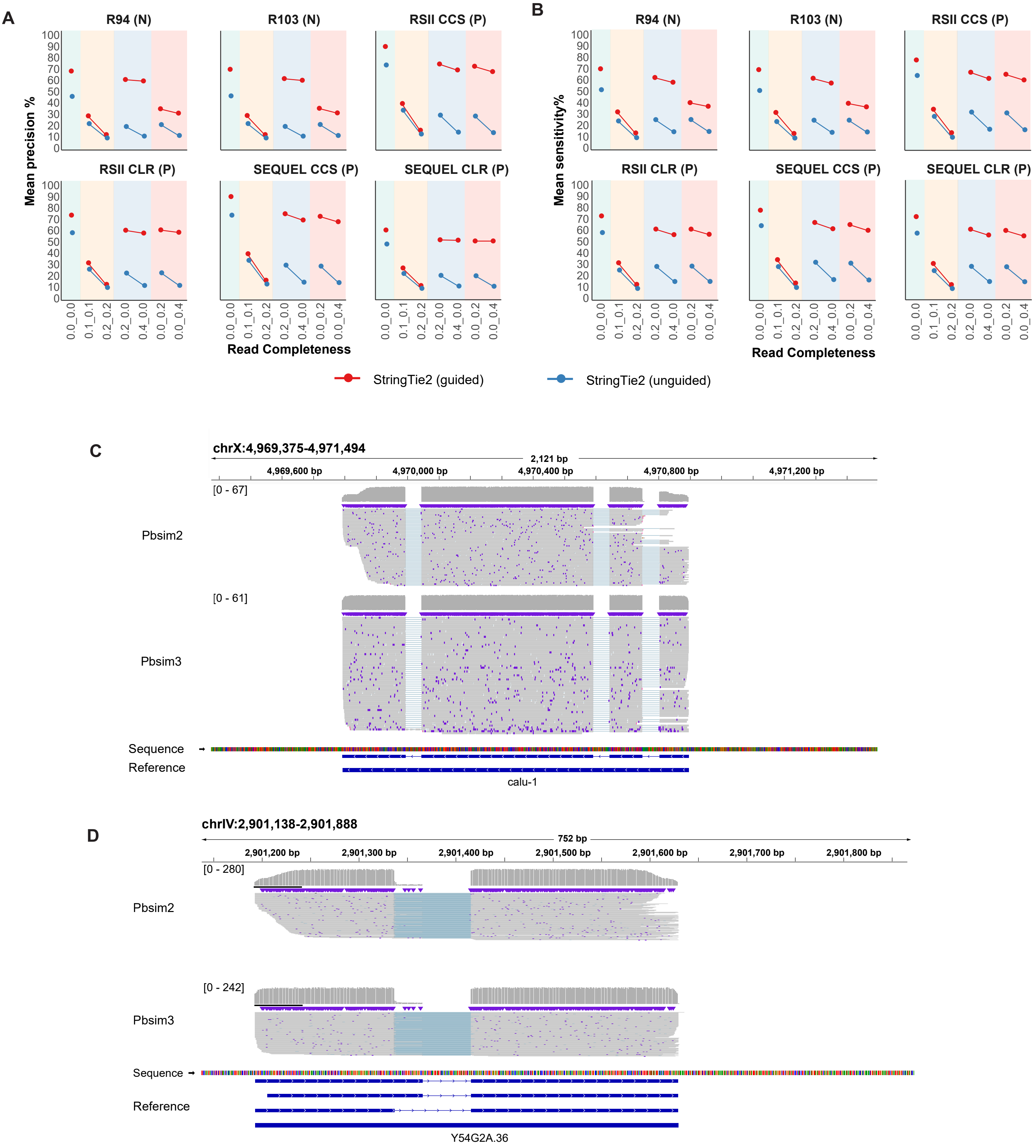


**Supplementary Figure 14. StringTie2 with "-R" Setting under Different Read Completeness Simulation Scenarios and IGV Screenshots showing the Difference between Reads Generated by Pbsim2 and Pbsim3.**
**A, B.** Mean precision (A) and sensitivity (B) for both the guided and unguided modes of StringTie2 with the "-R" parameter under different simulated datasets with completeness levels of 0.0_0.0: 100% complete, 0.1_0.1: 10% truncated from both ends; 0.2_0.2: 20% truncated from both ends; 0.2_0.0: 20% truncated from 5' end; 0.4_0.0: 40% truncated from 5' end; 0.0_0.2: 20% truncated from 3' end; 0.0_0.4: 40% truncated from 3' end. (n=126 in total). N and P represent datasets generated from the Nanopore and Pacbio platforms, respectively. All values are denoted as mean. Standard deviation (SD) is provided in the Source Data file. Source data underlying A and B are provided as a Source Data file. **C, D**. IGV screenshots of alignment of reads generated by Pbsim2 (model=R103, depth=25) and Pbsim3 (model = SEQUEL CLR, depth = 25) at two example gene loci: calu-1 (C) and Y54G2A.36 (D).

**Supplementary Table 1. A comprehensive overview of the software included for benchmarking.**

| Software | Programming Language | Version | Reference | Guidance Required | Error Correction | Functions | Algorithm Overview |
|---|---|---|---|---|---|---|---|
| FLAMES | Python, C, C++ | 1. 0 | Tian et al., 2021[1] | YES | YES | Isoform detection from both bulk and single-cell RNA-seq LRS data. | It first groups reads with similar splice sites to obtain a raw isoform annotation, which is then compared against the reference annotation for splice site correction and collapsing. The raw reads are then realigned to the new reference sequences containing those polished transcripts using minimap2 and transcripts with few read supports will eventually be discarded. |
| FLAIR | Python | 1.5.0 | Tang et al., 2020[2] | YES/NO | YES/NO | Can also perform alignment, quantification, differential expression, and differential splicing analysis. | It first corrects splice junctions according to the reference annotation file or short-read sequences, then groups the corrected reads by junctions and performs collapsing, and finally performs the read-isoform assignment and filters out low-confidence transcripts by coverage. |
| Freddie | Python | 0.3.1 | Orabi et al., 2023[3] | NO | YES | Provide a GTF with detected isoforms. | It first partitions the aligned reads so that no isoform has reads present in different split sets. The exon boundaries are then identified by finding a set of segmentation breakpoints that are best supported by the input split-alignments. Similar reads are then clustered together and the isoforms are constructed by generating the consensus structure for each cluster. |

| | | | | | | | |
|---|---|---|---|---|---|---|---|
| StringTie2 | C++ | 2.2.1 | Kovaka et al., 2019[4] | YES/NO | YES | Provides identified isoforms with abundance in the GTF format. Able to merge multiple sets of transcripts into a non-redundant set. | It first corrects splice sites in the reads with high-error alignment rate according to those with low-error alignment rate, then builds an alternative splice graph for each gene based on which a flow network is constructed for the path with the heaviest coverage, transcripts are then assembled, the path will be removed from the graph and the coverage will be updated until no more transcripts can be extracted. |
| Bambu | R | 3.0.8 | Chen et al., 2023[5] | YES/NO | YES | For guided mode it provides a GTF extended with the novel discovered isoforms based on the reference annotation; for unguided modes the output includes solely constructed transcripts. Both modes can output isoform quantification results. | It first utilizes a probabilistic model to correct junction alignments, and corrected reads that employ the same splice junctions are consolidated into read classes. It then combines read classes from all samples and calculates a cross-sample NDR. Third, compatible transcripts are assigned to each read class allowing for imprecise matches, and finally isoform expression estimates are obtained with the Expectation-Maximization (EM) algorithm for each sample. |
| TALON | Python | 5.0 | Wyman et al., 2019[6] | YES | NO | Output a GTF containing identified isoforms and a TSV file of the abundance information. | It first labels potential internal priming events in reads, then classifies transcripts into known and novel and records abundance according to the reference annotation, and eventually filters out novel transcripts that are not reproducible or with internal priming. |
| TAMA | Python | b0.0.0 | Kuo et al., 2020[7] | NO | YES | Can also merge different sets | It first collapses reads in capped/non-capped mode according to the defined wobble |

| | | | | | | | |
|---|---|---|---|---|---|---|---|
| | | | | | | of transcripts and perform ORF/NMD prediction. | thresholds, then filters out the low-confidence splice junctions according to the splice junction ranking or the amount of mapping mismatch surrounding splice junctions or both. |
| UNAGI | C, C++ | 1.0.1 | Al Kadi et al., 2020[8] | NO | YES | Output the detected isoforms in BED format. | It first strands reads into two files, which are then mapped to (with minimap2) and corrected using the reference genome. The transcriptional unit boundaries are then identified according to the coverage landscape for gene annotation. Spliced genes are identified separately and transcripts with similar splicing sites are clustered together, and the longest ORF is also searched for in each annotated gene for isoform detection. The identified isoforms with low read support are filtered out. |
| IsoQuant | Python | 3.3.1 | Prjibelski et al., 2023[9] | YES/NO | YES | Can also perform alignment and quantification. | IsoQuant utilizes long-read spliced alignments to build an intron graph, which is then leveraged to create paths representing complete transcripts. In the presence of reference annotation, IsoQuant initially allocates reads to established isoforms using an inexact intron-chain matching algorithm, considering splice site shifts. These assignments are subsequently employed for quantifying reference transcripts and rectifying inaccuracies in splice junction detection and misalignments. |

**Supplementary References**


1. Tian, L. *et al.* Comprehensive characterization of single-cell full-length isoforms in human and mouse with long-read sequencing. *Genome Biol* **22**, 310 (2021).
2. Tang, A. D. *et al.* Full-length transcript characterization of SF3B1 mutation in chronic lymphocytic leukemia reveals downregulation of retained introns. *Nat Commun* **11**, 1438 (2020).
3. Orabi, B. *et al.* Freddie: annotation-independent detection and discovery of transcriptomic alternative splicing isoforms using long-read sequencing. *Nucleic Acids Res* **51**, e11 (2023).
4. Kovaka, S. *et al.* Transcriptome assembly from long-read RNA-seq alignments with StringTie2. *Genome Biolo* **20**, 278 (2019).
5. Chen, Y. *et al.* Context-aware transcript quantification from long-read RNA-seq data with Bambu. *Nat Methods* **20**, 1187–1195 (2023).
6. Wyman, D. *et al.* A technology-agnostic long-read analysis pipeline for transcriptome discovery and quantification. Preprint at https://doi.org/10.1101/672931 (2019).
7. Kuo, R. I. *et al.* Illuminating the dark side of the human transcriptome with long read transcript sequencing. *BMC Genomics* **21**, 751 (2020).
8. Al kadi, M. *et al.* UNAGI: an automated pipeline for nanopore full-length cDNA sequencing uncovers novel transcripts and isoforms in yeast. *Funct Integr Genomics* **20**, 523–536 (2020).
9. Prjibelski, A. D. *et al.* Accurate isoform discovery with IsoQuant using long reads. *Nat Biotechnol* **41**, 915–918 (2023).
