# Supplementary material for: Comprehensive assessment of mRNA isoform detection methods for long-read sequencing data
Source: Nat Commun. 2024 May 10;15:3972. doi: 10.1038/s41467-024-48117-3 (PMC11087464; doi:10.1038/s41467-024-48117-3)
Supplement: Supplementary file 3 — Description of Additional Supplementary Files [file 41467_2024_48117_MOESM3_ESM.pdf]

Description of Additional Supplementary Files

File Name: Supplementary Data 1

Description: Summary information of experimental datasets.

File Name: Supplementary Data 2

Description: Isoform classification results obtained based on different methods using sequin RNA datasets.

File Name: Supplementary Data 3

Description: Isoform classification results obtained based on different methods using experimental datasets.

File Name: Supplementary Data 4

Description: Information of qPCR validation of the DIU Gene *RPL39L*.

File Name: Supplementary Data 5

Description: Long-read and short-read RNA-seq datasets used for GMM model fitting.

File Name: Supplementary Data 6

Description: qPCR primers list for *RPL39L* three different isoforms expression detection.
