# Supplementary material for: Comprehensive assessment of mRNA isoform detection methods for long-read sequencing data
Source: Nat Commun. 2024 May 10;15:3972. doi: 10.1038/s41467-024-48117-3 (PMC11087464; doi:10.1038/s41467-024-48117-3)
Supplement: Supplementary file 10 — Reporting Summary [file 41467_2024_48117_MOESM10_ESM.pdf]

nature portfolio

Corresponding author(s): Wanlu Liu

Last updated by author(s): 2024/3/27

Reporting Summary

Nature Portfolio wishes to improve the reproducibility of the work that we publish. This form provides structure for consistency and transparency in reporting. For further information on Nature Portfolio policies, see our Editorial Policies and the Editorial Policy Checklist.

Statistics

For all statistical analyses, confirm that the following items are present in the figure legend, table legend, main text, or Methods section.

n/a	Confirmed	
☐	☒	The exact sample size (*n*) for each experimental group/condition, given as a discrete number and unit of measurement
☐	☒	A statement on whether measurements were taken from distinct samples or whether the same sample was measured repeatedly
☐	☒	The statistical test(s) used AND whether they are one- or two-sided<br>*Only common tests should be described solely by name; describe more complex techniques in the Methods section.*
☐	☒	A description of all covariates tested
☒	☐	A description of any assumptions or corrections, such as tests of normality and adjustment for multiple comparisons
☐	☒	A full description of the statistical parameters including central tendency (e.g. means) or other basic estimates (e.g. regression coefficient) AND variation (e.g. standard deviation) or associated estimates of uncertainty (e.g. confidence intervals)
☐	☒	For null hypothesis testing, the test statistic (e.g. *F*, *t*, *r*) with confidence intervals, effect sizes, degrees of freedom and *P* value noted<br>*Give P values as exact values whenever suitable.*
☒	☐	For Bayesian analysis, information on the choice of priors and Markov chain Monte Carlo settings
☒	☐	For hierarchical and complex designs, identification of the appropriate level for tests and full reporting of outcomes
☒	☐	Estimates of effect sizes (e.g. Cohen's *d*, Pearson's *r*), indicating how they were calculated

Our web collection on statistics for biologists contains articles on many of the points above.

Software and code

Policy information about availability of computer code

Data collection	No software was used.
Data analysis	The software used for data analysis: IsoQuant (version 3.3.1); UNAGI (version 1.0.1); TAMA (version b0.0.0); TALON (version 5.0); Bambu (version 3.0.8); StringTie2 (version 2.2.1); Freddie (version 0.3.1); FLAIR (version 1.5.0); FLAMES (version 1.0); YASIM (version 3.2); minimap2 (version 2.17-r941); SAMtools (version 1.15.1); featureCounts (version 2.0.0); isoformSwitchAnalyzeR (version 1.8.0); GffRead (version 0.12.7); Salmon (version 1.8.0); FastQC (version 0.11.8); Cutadapt (version 2.9); STAR (version 2.7.1e); Python (version 3.8); psutil library (version 5.9); GffCompare (version 0.12.6). Code for YASIM can be found on GitHub via https://github.com/WanluLiuLab/yasim/ or on PYPI https://pypi.org/project/yasim/. Code for the profiler can be accessed via https://github.com/WanluLiuLab/labw_proc_profiler. Customized analysis code performed in this study can be found on GitHub via https://github.com/WanluLiuLab/2024_LRS_AS_Benchmark_Code.

For manuscripts utilizing custom algorithms or software that are central to the research but not yet described in published literature, software must be made available to editors and reviewers. We strongly encourage code deposition in a community repository (e.g. GitHub). See the Nature Portfolio guidelines for submitting code & software for further information.

Data

Policy information about availability of data

All manuscripts must include a data availability statement. This statement should provide the following information, where applicable:

- Accession codes, unique identifiers, or web links for publicly available datasets
- A description of any restrictions on data availability
- For clinical datasets or third party data, please ensure that the statement adheres to our policy

The datasets used in this study are available in National Centre for Biotechnology Information (NCBI) database under accession code SRR8568873 [https://www.ncbi.nlm.nih.gov/sra/?term=SRR8568873], SRR8568871 [https://www.ncbi.nlm.nih.gov/sra/?term=SRR8568871], SRR13762843 [https://www.ncbi.nlm.nih.gov/sra/?term=SRR13762843], and SRR13762841 [https://www.ncbi.nlm.nih.gov/sra/?term=SRR13762841]; SRR14630760 [https://www.ncbi.nlm.nih.gov/sra/?term=SRR14630760], SRR14630758 [https://www.ncbi.nlm.nih.gov/sra/?term=SRR14630758], SRR12800923 [https://www.ncbi.nlm.nih.gov/sra/?term=SRR12800923], SRR12800924 [https://www.ncbi.nlm.nih.gov/sra/?term=SRR12800924], SRR22522188 [https://www.ncbi.nlm.nih.gov/sra/?term=SRR22522188], SRR22522033 [https://www.ncbi.nlm.nih.gov/sra/?term=SRR22522033], SRR17960971 [https://www.ncbi.nlm.nih.gov/sra/?term=SRR17960971], SRR17960979 [https://www.ncbi.nlm.nih.gov/sra/?term=SRR17960979], SRR19257398 [https://www.ncbi.nlm.nih.gov/sra/?term=SRR19257398], and SRR19257401 [https://www.ncbi.nlm.nih.gov/sra/?term=SRR19257401]; ERR3588905 [https://www.ncbi.nlm.nih.gov/sra/?term=ERR3588905], and SRR13494726 [https://www.ncbi.nlm.nih.gov/sra/?term=SRR13494726]; SRR8929006 [https://www.ncbi.nlm.nih.gov/sra/?term=SRR8929006], SRR8929005 [https://www.ncbi.nlm.nih.gov/sra/?term=SRR8929005], SRR8929004 [https://www.ncbi.nlm.nih.gov/sra/?term=SRR8929004], SRR19055922 [https://www.ncbi.nlm.nih.gov/sra/?term=SRR19055922], and SRR19055924 [https://www.ncbi.nlm.nih.gov/sra/?term=SRR19055924]; SRR14073786 [https://www.ncbi.nlm.nih.gov/sra/?term=SRR14073786], SRR14073787 [https://www.ncbi.nlm.nih.gov/sra/?term=SRR14073787], SRR14073792 [https://www.ncbi.nlm.nih.gov/sra/?term=SRR14073792], and SRR14073793 [https://www.ncbi.nlm.nih.gov/sra/?term=SRR14073793] The four naïve and primed hESCs long-read RNA-seq datasets in this study have been deposited in the NCBI's Gene Expression Omnibus (GEO) under GEO Series accession number GSE227911 [https://www.ncbi.nlm.nih.gov/geo/query/acc.cgi?acc=GSE227911] The simulated datasets generated in this study are available upon request, as most public biological databases only accept experimental data. The size of those datasets limited us to deposit them in standard data sharing platforms. Please contact the corresponding author of this paper for access, and we will respond promptly.

Research involving human participants, their data, or biological material

Policy information about studies with human participants or human data. See also policy information about sex, gender (identity/presentation), and sexual orientation and race, ethnicity and racism.

Reporting on sex and gender	N.A.
Reporting on race, ethnicity, or other socially relevant groupings	N.A.
Population characteristics	N.A.
Recruitment	N.A.
Ethics oversight	N.A.

Note that full information on the approval of the study protocol must also be provided in the manuscript.

Field-specific reporting

Please select the one below that is the best fit for your research. If you are not sure, read the appropriate sections before making your selection.

☒ Life sciences ☐ Behavioural & social sciences ☐ Ecological, evolutionary & environmental sciences

For a reference copy of the document with all sections, see nature.com/documents/nr-reporting-summary-flat.pdf

Life sciences study design

All studies must disclose on these points even when the disclosure is negative.

Sample size	For the simulated datasets, we utilized a total of 456 datasets, each with 3 replicates under different conditions. This extensive set of simulated data was designed to encompass a wide range of scenarios and conditions that are commonly encountered in long-read sequencing experiments. Additionally, we included 16 publicly available sequins RNA datasets and 25 publicly available experimental RNA-seq datasets to provide real-world validation and comparison to the simulated data. For the simulated datasets, the number of replicates (3 per condition) was chosen to balance statistical power with computational feasibility, ensuring reliable estimates of performance metrics for the evaluated software. Similarly, the selection of 16 sequins RNA datasets and 25 experimental RNA-seq datasets was based on the availability of high-quality, publicly accessible data that represented a diverse range of biological conditions and experimental setups. The sample size for the RT-qPCR validation experiments, which involved three biological replicates, was determined based on standard practices in molecular biology research. Three replicates were chosen to ensure statistical reliability and to capture potential variability between biological samples while also considering practical constraints such as resources and time.

	No specific statistical methods were used to predetermine sample sizes for the RT-qPCR experiments as the choice of three replicates is a commonly accepted practice in biological research for ensuring reproducibility and reliability of results.
Data exclusions	No data were excluded for the analysis.
Replication	All the codes used for analysis have been provided, based on which the same result can be obtained from the specific given input dataset.
Randomization	Randomization was not relevant to this study since each computational tool included for benchmarking processed the same groups of data.
Blinding	Regarding the data collection phase, blinding was not relevant as all datasets used in our benchmark study were sourced from public databases, where the data were openly available and not subject to manipulation or bias during collection. The primary purpose of our study was to evaluate the performance of computational tools using these publicly available datasets. Therefore, blinding of data collection was not deemed necessary as the datasets themselves were inherently unbiased and impartial. Similarly, blinding was not considered relevant during the data analysis phase. The benchmarking process involved running computational tools on the collected datasets and assessing their performance based on predefined metrics. The analysis was conducted in a systematic and objective manner, without subjective interpretation or influence from the researchers. Additionally, the performance metrics used for evaluation were quantitative and objective, minimizing the potential for bias or subjective judgment.

Reporting for specific materials, systems and methods

We require information from authors about some types of materials, experimental systems and methods used in many studies. Here, indicate whether each material, system or method listed is relevant to your study. If you are not sure if a list item applies to your research, read the appropriate section before selecting a response.

Materials & experimental systems

n/a	Involved in the study
☒	☐ Antibodies
☐	☒ Eukaryotic cell lines
☒	☐ Palaeontology and archaeology
☒	☐ Animals and other organisms
☒	☐ Clinical data
☒	☐ Dual use research of concern
☒	☐ Plants

Methods

n/a	Involved in the study
☒	☐ ChIP-seq
☒	☐ Flow cytometry
☒	☐ MRI-based neuroimaging

Eukaryotic cell lines

Policy information about cell lines and Sex and Gender in Research

Cell line source(s)	H1 hESC cell line (Wicell Research Institute, Inc., WA01-pcbc) were applied in this research.
Authentication	The cell line were not authenticated.
Mycoplasma contamination	Both naïve and primed hESCs were regularly tested for mycoplasma contamination, and all tests showed negative results.
Commonly misidentified lines (See ICLAC register)	No commonly misidentified cell lines were used in the study.
